# 6-Chloro-7-methyl-4-oxo-4*H*-chromene-3-carbaldehyde

**DOI:** 10.1107/S1600536814014226

**Published:** 2014-06-21

**Authors:** Yoshinobu Ishikawa

**Affiliations:** a School of Pharmaceutical Sciences, University of Shizuoka, 52-1 Yada, Suruga-ku, Shizuoka 422-8526, Japan

**Keywords:** crystal structure

## Abstract

In the title compound, C_11_H_7_ClO_3_, a chlorinated and methyl­ated 3-formyl­chromone derivative, the non-H atoms are essentially coplanar (r.m.s. deviation = 0.0670 Å), with the largest deviation from the least-squares plane [0.2349 (17) Å] being for the pyran carbonyl O atom. In the crystal, mol­ecules are linked through π–π stacking inter­actions along the *a* axis [centroid–centroid distance between the pyran rings = 3.824 (6) Å] and two stacks are connected by type I halogen–halogen inter­actions between the Cl atoms [Cl⋯Cl = 3.397 (3) Å].

## Related literature   

For related structures, see: Ishikawa & Motohashi (2013 ▶); Ishikawa (2014 ▶). For halogen bonding, see: Auffinger *et al.* (2004 ▶); Metrangolo *et al.* (2005 ▶); Wilcken *et al.* (2013 ▶); Sirimulla *et al.* (2013 ▶). For halogen–halogen inter­actions, see: Metrangolo & Resnati (2014 ▶); Mukherjee & Desiraju (2014 ▶).
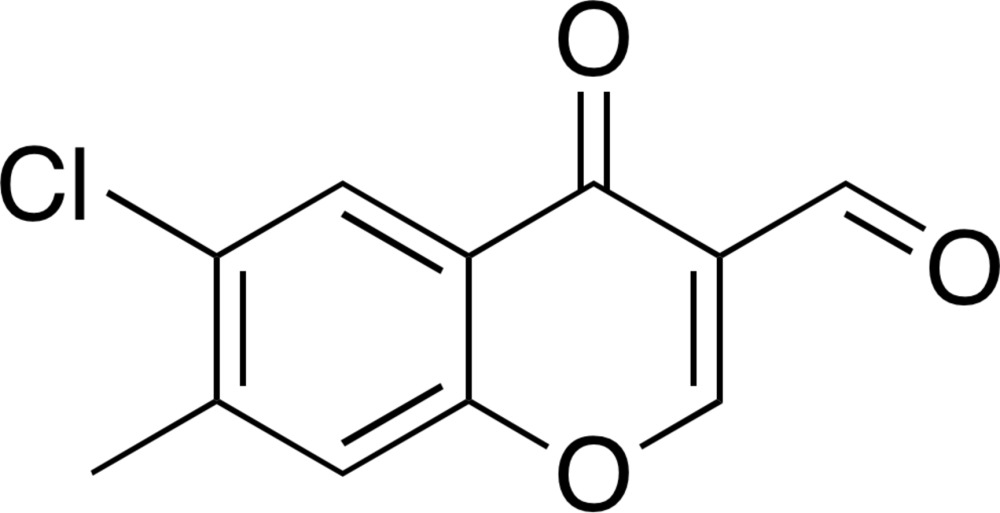


## Experimental   

### 

#### Crystal data  

C_11_H_7_ClO_3_
*M* *_r_* = 222.63
Triclinic, 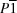

*a* = 3.824 (6) Å
*b* = 6.111 (9) Å
*c* = 19.962 (10) Å
α = 81.83 (7)°
β = 88.82 (7)°
γ = 87.04 (12)°
*V* = 461.1 (10) Å^3^
*Z* = 2
Mo *K*α radiation
μ = 0.39 mm^−1^
*T* = 100 K
0.45 × 0.20 × 0.10 mm

#### Data collection  

Rigaku AFC-7R diffractometer
2677 measured reflections
2092 independent reflections
1784 reflections with *F* ^2^ > 2σ(*F* ^2^)
*R*_int_ = 0.076
3 standard reflections every 150 reflections intensity decay: −0.3%

#### Refinement  

*R*[*F* ^2^ > 2σ(*F* ^2^)] = 0.034
*wR*(*F* ^2^) = 0.094
*S* = 1.08
2092 reflections
137 parameters
H-atom parameters constrained
Δρ_max_ = 0.43 e Å^−3^
Δρ_min_ = −0.44 e Å^−3^

### 

Data collection: *WinAFC Diffractometer Control Software* (Rigaku, 1999 ▶); cell refinement: *WinAFC Diffractometer Control Software*; data reduction: *WinAFC Diffractometer Control Software*; program(s) used to solve structure: *SIR2008* (Burla *et al.*, 2007 ▶); program(s) used to refine structure: *SHELXL97* (Sheldrick, 2008 ▶); molecular graphics: *CrystalStructure* (Rigaku, 2010 ▶); software used to prepare material for publication: *CrystalStructure*.

## Supplementary Material

Crystal structure: contains datablock(s) General, I. DOI: 10.1107/S1600536814014226/tk5320sup1.cif

Structure factors: contains datablock(s) I. DOI: 10.1107/S1600536814014226/tk5320Isup2.hkl

Supporting information file. DOI: 10.1107/S1600536814014226/tk5320Isup3.cml

CCDC reference: 1008807

Additional supporting information:  crystallographic information; 3D view; checkCIF report

## Figures and Tables

**Fig. 1.**
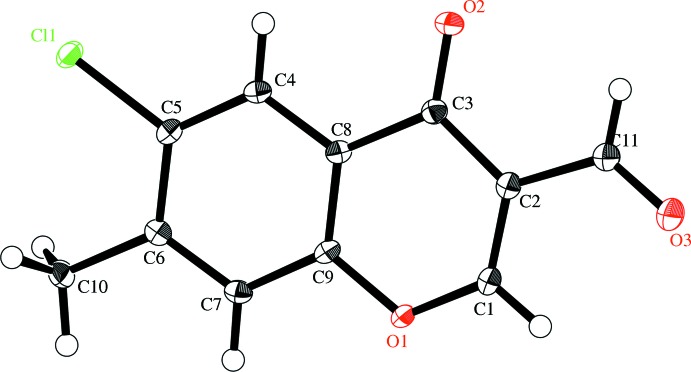
The molecular structure of the title compound, with displacement ellipsoids drawn at the 50% probability level. Hydrogen atoms are shown as small spheres of arbitrary radius.

**Fig. 2.**
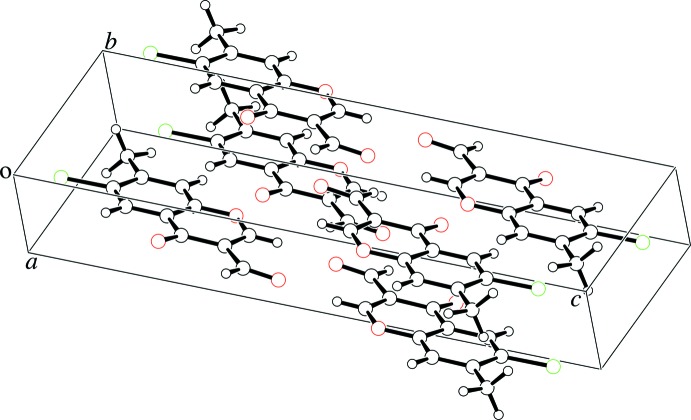
A packing view of the title compound.

**Fig. 3.**
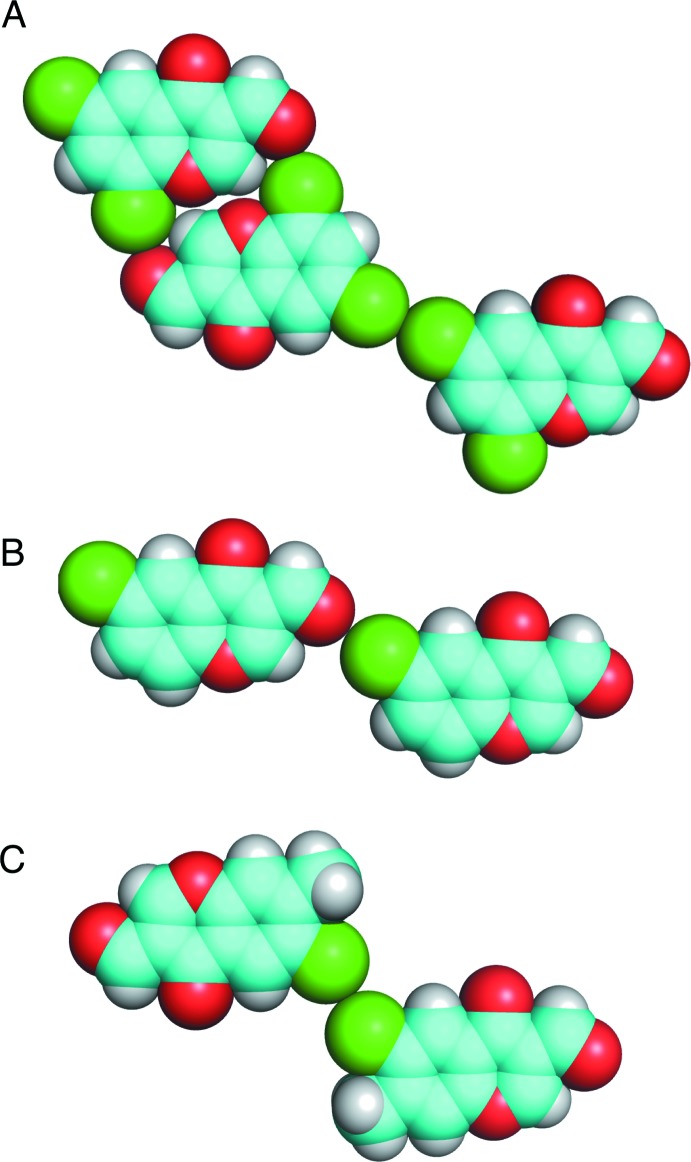
Sphere models of the crystal structures of 6,8-dichloro-4-oxochromene-3-carbaldehyde (top), 6-chloro-4-oxo-4H-chromene-3-carbaldehyde (middle), and the title compound (bottom).

## supplementary crystallographic information

### S1. Structural commentary

Halogen bonding and halogen···halogen inter­actions have recently attracted much attention in medicinal chemistry, chemical biology, supra­molecular chemistry, and crystal engineering (Auffinger *et al.*, 2004, Metrangolo *et al*., 2005, Wilcken *et al.*, 2013, Sirimulla *et al.*, 2013, Mukherjee & Desiraju, 2014, Metrangolo & Resnati, 2014). We have recently reported the crystal structures of halogenated 3-formyl­chromone derivatives 6,8-di­chloro-4-oxochromene-3-carbaldehyde (Ishikawa & Motohashi, 2013) and 6-chloro-4-oxo-4*H*-chromene-3-carbaldehyde (Ishikawa, 2014). Both halogen bonding between the formyl oxygen atom and the chlorine atom at 8-position and type I halogen···halogen inter­action between the chlorine atoms at 6-position are observed in 6,8-di­chloro-4-oxochromene-3-carbaldehyde (Fig. 3, (top). On the other hand, a van der Waals contact between the formyl oxygen atom and the chlorine atom at 6-position is found in 6-chloro-4-oxo-4*H*-chromene-3-carbaldehyde (Fig. 3, middle). As part of our inter­est in these types of chemical bonding, we herein report the crystal structure of a monochlorinated and methyl­ated 3-formyl­chromone derivative 6-chloro-7-methyl-4-oxo-4*H*-chromene-3-carbaldehyde. The objective of this study is to reveal the inductive effect of the vicinal electron-donating group on the chlorine atom at 6-position and its inter­action mode.

The mean deviation of the least-square plane for the non-hydrogen atoms is 0.0670 Å, and the largest deviation is 0.2349 (17) Å for O3 (Fig. 1).

In the crystal, the molecules are stacked with the translation-symmetry equivalent^i^ [centroid–centroid distance between the pyran rings = 3.824 (6) Å, i: *x* + 1, *y*, *z*], as shown in Fig. 2. In addition, a type I halogen···halogen inter­action is observed between the chlorine atoms at 6-position [Cl1···Cl1^ii^ = 3.397 (3) Å, C5–Cl1–Cl1^ii^ = 148.41 (7)°, ii: –*x*, –*y*, –*z*], as shown in Fig. 3 (bottom). Thus, a contact between the formyl oxygen atom and the chlorine atom at 6-position is not observed in the title compound. The chemical nature of the chlorine atom at 6-position in the title compound should be similar to that of the chlorine one at 6-position in 6,8-di­chloro-4-oxochromene-3-carbaldehyde.

### S2. Synthesis and crystallization

Single crystals suitable for X-ray diffraction were obtained by slow evaporation of an aceto­nitrile solution of the commercially available title compound at room temperature.

### S3. Refinement

The C(*sp*^2^)-bound hydrogen atoms were placed in geometrical positions [C—H 0.95 Å, *U*_iso_(H) = 1.2*U*_eq_(C)], and refined using a riding model. One reflection (0 0 20) was omitted because of systematic error.

### Crystal data

**Table:**

C_11_H_7_ClO_3_	*Z* = 2
*M**_r_* = 222.63	*F*(000) = 228.00
Triclinic, *P*1	*D*_x_ = 1.603 Mg m^−^^3^
Hall symbol: -P 1	Mo *K*α radiation, λ = 0.71069 Å
*a* = 3.824 (6) Å	Cell parameters from 25 reflections
*b* = 6.111 (9) Å	θ = 15.1–17.5°
*c* = 19.962 (10) Å	µ = 0.39 mm^−^^1^
α = 81.83 (7)°	*T* = 100 K
β = 88.82 (7)°	Plate, colorless
γ = 87.04 (12)°	0.45 × 0.20 × 0.10 mm
*V* = 461.1 (10) Å^3^	

### Data collection

**Table:**

Rigaku AFC-7R diffractometer	θ_max_ = 27.5°
ω–2θ scans	*h* = −4→2
2677 measured reflections	*k* = −7→7
2092 independent reflections	*l* = −25→25
1784 reflections with *F*^2^ > 2σ(*F*^2^)	3 standard reflections every 150 reflections
*R*_int_ = 0.076	intensity decay: −0.3%

### Refinement

**Table:**

Refinement on *F*^2^	Secondary atom site location: difference Fourier map
*R*[*F*^2^ > 2σ(*F*^2^)] = 0.034	Hydrogen site location: inferred from neighbouring sites
*wR*(*F*^2^) = 0.094	H-atom parameters constrained
*S* = 1.08	*w* = 1/[σ^2^(*F*_o_^2^) + (0.0479*P*)^2^ + 0.2265*P*] where *P* = (*F*_o_^2^ + 2*F*_c_^2^)/3
2092 reflections	(Δ/σ)_max_ < 0.001
137 parameters	Δρ_max_ = 0.43 e Å^−^^3^
0 restraints	Δρ_min_ = −0.44 e Å^−^^3^
Primary atom site location: structure-invariant direct methods	

### Special details

**Table:**

Refinement. Refinement was performed using all reflections. The weighted *R*-factor (*wR*) and goodness of fit (*S*) are based on *F*^2^. *R*-factor (gt) are based on *F*. The threshold expression of *F*^2^ > 2.0 σ(*F*^2^) is used only for calculating *R*-factor (gt).

### Fractional atomic coordinates and isotropic or equivalent isotropic displacement parameters (Å^2^)

**Table:**

	*x*	*y*	*z*	*U*_iso_*/*U*_eq_	
Cl1	0.17920 (11)	0.14364 (6)	0.056454 (19)	0.01599 (13)	
O1	0.4150 (4)	0.24855 (18)	0.33907 (6)	0.0147 (3)	
O2	−0.0788 (4)	−0.30118 (19)	0.30226 (6)	0.0175 (3)	
O3	0.2039 (4)	−0.2523 (3)	0.49681 (6)	0.0257 (3)	
C1	0.3352 (5)	0.0873 (3)	0.38908 (8)	0.0149 (4)	
C2	0.1817 (5)	−0.1014 (3)	0.38028 (8)	0.0143 (4)	
C3	0.0832 (5)	−0.1417 (3)	0.31299 (8)	0.0125 (3)	
C4	0.1395 (4)	0.0114 (3)	0.19037 (8)	0.0124 (3)	
C5	0.2401 (4)	0.1770 (3)	0.14057 (8)	0.0125 (3)	
C6	0.3901 (4)	0.3707 (3)	0.15567 (8)	0.0123 (3)	
C7	0.4419 (4)	0.3892 (3)	0.22300 (8)	0.0131 (3)	
C8	0.1912 (4)	0.0316 (3)	0.25829 (8)	0.0117 (3)	
C9	0.3461 (4)	0.2198 (3)	0.27341 (8)	0.0122 (3)	
C10	0.4919 (5)	0.5510 (3)	0.10037 (9)	0.0158 (4)	
C11	0.1133 (5)	−0.2676 (3)	0.43982 (9)	0.0185 (4)	
H1	0.3904	0.1068	0.4340	0.0179*	
H2	0.0357	−0.1156	0.1786	0.0149*	
H3	0.5426	0.5171	0.2349	0.0157*	
H4A	0.6025	0.6673	0.1203	0.0190*	
H5B	0.2824	0.6139	0.0758	0.0190*	
H6C	0.6571	0.4893	0.0690	0.0190*	
H7	−0.0088	−0.3940	0.4331	0.0222*	

### Atomic displacement parameters (Å^2^)

**Table:**

	*U*^11^	*U*^22^	*U*^33^	*U*^12^	*U*^13^	*U*^23^
Cl1	0.0226 (3)	0.0153 (2)	0.0106 (2)	−0.00165 (14)	−0.00239 (13)	−0.00318 (13)
O1	0.0210 (6)	0.0146 (6)	0.0093 (6)	−0.0054 (5)	−0.0015 (5)	−0.0026 (4)
O2	0.0222 (7)	0.0151 (6)	0.0160 (6)	−0.0074 (5)	−0.0018 (5)	−0.0024 (5)
O3	0.0382 (8)	0.0262 (7)	0.0129 (7)	−0.0108 (6)	−0.0028 (6)	0.0009 (5)
C1	0.0178 (8)	0.0169 (8)	0.0102 (8)	−0.0016 (6)	−0.0000 (6)	−0.0019 (6)
C2	0.0161 (8)	0.0148 (8)	0.0121 (8)	−0.0014 (6)	0.0000 (6)	−0.0018 (6)
C3	0.0119 (7)	0.0129 (7)	0.0130 (8)	−0.0000 (6)	−0.0002 (6)	−0.0030 (6)
C4	0.0123 (8)	0.0113 (7)	0.0142 (8)	−0.0005 (6)	−0.0012 (6)	−0.0039 (6)
C5	0.0125 (8)	0.0140 (7)	0.0116 (7)	0.0012 (6)	−0.0012 (6)	−0.0037 (6)
C6	0.0101 (7)	0.0113 (7)	0.0151 (8)	0.0011 (6)	−0.0000 (6)	−0.0015 (6)
C7	0.0128 (8)	0.0108 (7)	0.0160 (8)	−0.0011 (6)	−0.0004 (6)	−0.0032 (6)
C8	0.0119 (8)	0.0110 (7)	0.0125 (8)	−0.0000 (6)	−0.0016 (6)	−0.0026 (6)
C9	0.0124 (8)	0.0136 (7)	0.0114 (8)	0.0001 (6)	−0.0017 (6)	−0.0044 (6)
C10	0.0198 (8)	0.0120 (7)	0.0152 (8)	−0.0021 (6)	0.0010 (6)	0.0001 (6)
C11	0.0237 (9)	0.0177 (8)	0.0143 (8)	−0.0054 (7)	−0.0004 (7)	−0.0015 (6)

### Geometric parameters (Å, º)

**Table:**

Cl1—C5	1.7418 (19)	C6—C7	1.384 (3)
O1—C1	1.341 (3)	C6—C10	1.505 (3)
O1—C9	1.379 (3)	C7—C9	1.395 (3)
O2—C3	1.228 (3)	C8—C9	1.394 (3)
O3—C11	1.214 (3)	C1—H1	0.950
C1—C2	1.355 (3)	C4—H2	0.950
C2—C3	1.460 (3)	C7—H3	0.950
C2—C11	1.478 (3)	C10—H4A	0.980
C3—C8	1.477 (3)	C10—H5B	0.980
C4—C5	1.378 (3)	C10—H6C	0.980
C4—C8	1.398 (3)	C11—H7	0.950
C5—C6	1.414 (3)		
			
Cl1···C10	3.061 (5)	O3···H7^iv^	3.3646
O1···C3	2.874 (5)	O3···H7^viii^	2.5400
O2···C1	3.580 (5)	C1···H7^ii^	3.5865
O2···C4	2.865 (4)	C2···H1^vii^	3.4323
O2···C11	2.895 (3)	C2···H7^iv^	3.5942
O3···C1	2.825 (4)	C3···H3^v^	3.5438
C1···C7	3.584 (4)	C3···H3^vi^	3.2093
C1···C8	2.751 (3)	C4···H2^iv^	3.4855
C2···C9	2.772 (4)	C4···H3^vi^	3.3323
C4···C7	2.797 (5)	C4···H4A^v^	3.4637
C5···C9	2.742 (3)	C4···H4A^vi^	3.1405
C6···C8	2.821 (4)	C4···H5B^vi^	3.5800
Cl1···Cl1^i^	3.397 (3)	C5···H2^iv^	3.4899
O1···O2^ii^	3.259 (5)	C5···H4A^vi^	3.4169
O1···O2^iii^	3.432 (5)	C5···H6C^vii^	3.0939
O1···C2^iv^	3.578 (6)	C6···H2^ii^	3.4420
O1···C3^iv^	3.487 (6)	C6···H4A^vii^	3.4668
O2···O1^v^	3.432 (5)	C6···H6C^vii^	3.3186
O2···O1^vi^	3.259 (5)	C7···H2^ii^	3.3414
O2···C2^vii^	3.452 (5)	C7···H3^vii^	3.4983
O2···C3^vii^	3.313 (6)	C8···H3^vi^	3.4446
O2···C7^v^	3.282 (4)	C9···H3^vii^	3.5296
O2···C7^vi^	3.222 (5)	C10···H2^ii^	3.1715
O2···C8^vii^	3.422 (6)	C10···H2^iii^	3.5202
O2···C9^vi^	3.389 (5)	C10···H4A^vii^	3.4653
O3···O3^viii^	3.462 (5)	C10···H5B^iv^	3.0874
O3···O3^ix^	3.400 (5)	C10···H6C^vii^	3.3170
O3···C1^ix^	3.271 (4)	C10···H6C^xii^	3.4850
O3···C1^x^	3.212 (4)	C11···H1^vii^	3.4892
O3···C11^viii^	3.306 (5)	C11···H1^ix^	3.3664
C1···O3^ix^	3.271 (4)	C11···H1^x^	3.4654
C1···O3^x^	3.212 (4)	C11···H7^iv^	3.4101
C1···C2^iv^	3.390 (6)	C11···H7^viii^	3.0703
C1···C3^iv^	3.530 (5)	H1···O3^ix^	2.8065
C2···O1^vii^	3.578 (6)	H1···O3^x^	2.3848
C2···O2^iv^	3.452 (5)	H1···C2^iv^	3.4323
C2···C1^vii^	3.390 (6)	H1···C11^iv^	3.4892
C3···O1^vii^	3.487 (6)	H1···C11^ix^	3.3664
C3···O2^iv^	3.313 (6)	H1···C11^x^	3.4654
C3···C1^vii^	3.530 (5)	H1···H1^x^	2.8906
C3···C9^vii^	3.522 (6)	H1···H7^ii^	3.3381
C4···C6^vii^	3.537 (6)	H2···C4^vii^	3.4855
C4···C7^vii^	3.548 (6)	H2···C5^vii^	3.4899
C5···C6^vii^	3.424 (6)	H2···C6^vi^	3.4420
C5···C10^vii^	3.599 (6)	H2···C7^vi^	3.3414
C6···C4^iv^	3.537 (6)	H2···C10^v^	3.5202
C6···C5^iv^	3.424 (6)	H2···C10^vi^	3.1715
C7···O2^ii^	3.222 (5)	H2···H3^v^	3.0807
C7···O2^iii^	3.282 (4)	H2···H3^vi^	2.9969
C7···C4^iv^	3.548 (6)	H2···H4A^v^	2.5702
C7···C8^iv^	3.533 (6)	H2···H4A^vi^	2.8020
C8···O2^iv^	3.422 (6)	H2···H5B^vi^	2.9247
C8···C7^vii^	3.533 (6)	H3···O2^ii^	2.9512
C8···C9^vii^	3.396 (6)	H3···O2^iii^	2.4107
C9···O2^ii^	3.389 (5)	H3···C3^ii^	3.2093
C9···C3^iv^	3.522 (6)	H3···C3^iii^	3.5438
C9···C8^iv^	3.396 (6)	H3···C4^ii^	3.3323
C10···C5^iv^	3.599 (6)	H3···C7^iv^	3.4983
C11···O3^viii^	3.306 (5)	H3···C8^ii^	3.4446
C11···C11^viii^	3.581 (5)	H3···C9^iv^	3.5296
Cl1···H2	2.7736	H3···H2^ii^	2.9969
Cl1···H5B	3.0008	H3···H2^iii^	3.0807
Cl1···H6C	2.9039	H4A···Cl1^ii^	3.3558
O1···H3	2.5156	H4A···C4^ii^	3.1405
O2···H2	2.6042	H4A···C4^iii^	3.4637
O2···H7	2.6084	H4A···C5^ii^	3.4169
O3···H1	2.4980	H4A···C6^iv^	3.4668
C1···H7	3.2820	H4A···C10^iv^	3.4653
C3···H1	3.2957	H4A···H2^ii^	2.8020
C3···H2	2.6739	H4A···H2^iii^	2.5702
C3···H7	2.6867	H4A···H5B^iv^	2.7486
C5···H3	3.2635	H5B···Cl1^ii^	3.2097
C5···H4A	3.3351	H5B···Cl1^xi^	3.3310
C5···H5B	2.8070	H5B···Cl1^xii^	3.5195
C5···H6C	2.7698	H5B···C4^ii^	3.5800
C6···H2	3.2984	H5B···C10^vii^	3.0874
C7···H4A	2.5561	H5B···H2^ii^	2.9247
C7···H5B	3.1190	H5B···H4A^vii^	2.7486
C7···H6C	3.1491	H5B···H6C^vii^	2.5591
C8···H3	3.2892	H5B···H6C^xii^	3.0471
C9···H1	3.1874	H6C···Cl1^iv^	2.8645
C9···H2	3.2616	H6C···Cl1^xii^	3.1901
C10···H3	2.6736	H6C···C5^iv^	3.0939
C11···H1	2.5574	H6C···C6^iv^	3.3186
H1···H7	3.4925	H6C···C10^iv^	3.3170
H3···H4A	2.3514	H6C···C10^xii^	3.4850
H3···H5B	3.3096	H6C···H5B^iv^	2.5591
H3···H6C	3.3584	H6C···H5B^xii^	3.0471
Cl1···H4A^vi^	3.3558	H6C···H6C^xii^	3.0105
Cl1···H5B^vi^	3.2097	H7···O1^vi^	3.4049
Cl1···H5B^xi^	3.3310	H7···O3^vii^	3.3646
Cl1···H5B^xii^	3.5195	H7···O3^viii^	2.5400
Cl1···H6C^vii^	2.8645	H7···C1^vi^	3.5865
Cl1···H6C^xii^	3.1901	H7···C2^vii^	3.5942
O1···H7^ii^	3.4049	H7···C11^vii^	3.4101
O2···H3^v^	2.4107	H7···C11^viii^	3.0703
O2···H3^vi^	2.9512	H7···H1^vi^	3.3381
O3···H1^ix^	2.8065	H7···H7^viii^	2.7996
O3···H1^x^	2.3848		
			
C1—O1—C9	118.32 (15)	O1—C9—C7	116.26 (16)
O1—C1—C2	124.81 (17)	O1—C9—C8	121.84 (15)
C1—C2—C3	120.85 (15)	C7—C9—C8	121.90 (17)
C1—C2—C11	119.27 (17)	O3—C11—C2	123.93 (18)
C3—C2—C11	119.87 (17)	O1—C1—H1	117.595
O2—C3—C2	123.93 (15)	C2—C1—H1	117.592
O2—C3—C8	122.62 (17)	C5—C4—H2	120.213
C2—C3—C8	113.45 (16)	C8—C4—H2	120.226
C5—C4—C8	119.56 (17)	C6—C7—H3	120.052
Cl1—C5—C4	118.24 (15)	C9—C7—H3	120.055
Cl1—C5—C6	119.54 (13)	C6—C10—H4A	109.463
C4—C5—C6	122.22 (17)	C6—C10—H5B	109.471
C5—C6—C7	117.93 (15)	C6—C10—H6C	109.471
C5—C6—C10	121.19 (17)	H4A—C10—H5B	109.473
C7—C6—C10	120.87 (16)	H4A—C10—H6C	109.475
C6—C7—C9	119.89 (17)	H5B—C10—H6C	109.474
C3—C8—C4	121.00 (16)	O3—C11—H7	118.044
C3—C8—C9	120.54 (16)	C2—C11—H7	118.029
C4—C8—C9	118.46 (15)		
			
C1—O1—C9—C7	−178.36 (12)	H2—C4—C5—C6	178.9
C1—O1—C9—C8	1.3 (2)	H2—C4—C8—C3	−0.6
C9—O1—C1—C2	−1.9 (3)	H2—C4—C8—C9	179.6
C9—O1—C1—H1	178.1	Cl1—C5—C6—C7	−178.56 (10)
O1—C1—C2—C3	−1.1 (3)	Cl1—C5—C6—C10	1.2 (2)
O1—C1—C2—C11	179.01 (13)	C4—C5—C6—C7	1.3 (3)
H1—C1—C2—C3	178.9	C4—C5—C6—C10	−178.89 (13)
H1—C1—C2—C11	−1.0	C5—C6—C7—C9	−0.1 (3)
C1—C2—C3—O2	−174.55 (15)	C5—C6—C7—H3	179.9
C1—C2—C3—C8	4.3 (2)	C5—C6—C10—H4A	−176.9
C1—C2—C11—O3	−4.2 (3)	C5—C6—C10—H5B	63.1
C1—C2—C11—H7	175.8	C5—C6—C10—H6C	−56.9
C3—C2—C11—O3	175.88 (15)	C7—C6—C10—H4A	2.8
C3—C2—C11—H7	−4.1	C7—C6—C10—H5B	−117.1
C11—C2—C3—O2	5.3 (3)	C7—C6—C10—H6C	122.8
C11—C2—C3—C8	−175.85 (13)	C10—C6—C7—C9	−179.90 (13)
O2—C3—C8—C4	−5.7 (3)	C10—C6—C7—H3	0.1
O2—C3—C8—C9	174.08 (14)	C6—C7—C9—O1	178.34 (13)
C2—C3—C8—C4	175.51 (13)	C6—C7—C9—C8	−1.3 (3)
C2—C3—C8—C9	−4.8 (2)	H3—C7—C9—O1	−1.7
C5—C4—C8—C3	179.37 (13)	H3—C7—C9—C8	178.7
C5—C4—C8—C9	−0.4 (3)	C3—C8—C9—O1	2.2 (3)
C8—C4—C5—Cl1	178.80 (12)	C3—C8—C9—C7	−178.17 (13)
C8—C4—C5—C6	−1.1 (3)	C4—C8—C9—O1	−178.08 (13)
H2—C4—C5—Cl1	−1.2	C4—C8—C9—C7	1.6 (3)

Symmetry codes: (i) −*x*, −*y*, −*z*; (ii) *x*, *y*+1, *z*; (iii) *x*+1, *y*+1, *z*; (iv) *x*+1, *y*, *z*; (v) *x*−1, *y*−1, *z*; (vi) *x*, *y*−1, *z*; (vii) *x*−1, *y*, *z*; (viii) −*x*, −*y*−1, −*z*+1; (ix) −*x*, −*y*, −*z*+1; (x) −*x*+1, −*y*, −*z*+1; (xi) −*x*, −*y*+1, −*z*; (xii) −*x*+1, −*y*+1, −*z*.
